# Smartphone-Related Neck Pain: A Study Among Saudi Residents in the Eastern Province

**DOI:** 10.7759/cureus.68457

**Published:** 2024-09-02

**Authors:** Anas E Ahmed, Mohammed E Mojiri, Osama A Mobarki, Osama A Suwaid, Hussam M Kariri, Abdullatif Z Suwaydi, Fahad Y Moafa, Yazeed A Alhelali, Turki N Abo Sarhad, Omar S Al Obaid, Abdulaziz S Almutlaq, Jenan A Alhussain, Kamlah I Samkari, Naif A Gharwi, Fatimah M Akkam

**Affiliations:** 1 Department of Community Medicine, Jazan University, Jazan, SAU; 2 College of Medicine, Jazan University, Jazan, SAU; 3 College of Applied Medical Sciences, Jazan University, Jazan, SAU; 4 Department of Orthopedics, Aseer Central Hospital, Abha, SAU; 5 College of Medicine, King Khalid University, Abha, SAU; 6 College of Medicine, King Faisal University, Al-Ahsa, SAU; 7 College of Medicine, Almaarefa University, Riyadh, SAU

**Keywords:** health risks, phone use patterns, musculoskeletal health, mobile phone usage, eastern province, saudi arabia, neck pain, smartphone

## Abstract

Background: The pervasive use of mobile phones has raised concerns about their impact on musculoskeletal health, particularly neck pain. This issue is notably relevant in the Eastern Province of Saudi Arabia, where high mobile phone usage intersects with demographic diversity. While extensive phone use has been linked to neck pain and other musculoskeletal disorders globally, specific data on this issue in the Eastern Province are limited. This study addresses this gap by examining phone use patterns, neck positions, and associated symptoms in the region.

Methods: Using an online, self-administered survey, this cross-sectional study investigated the relationship between phone use and neck pain in the Eastern Province of Saudi Arabia. Participants aged 18 years and older were recruited via social media, community groups, and university networks. The survey collected data on demographics, phone use patterns, neck positions, awareness of health risks, and pain symptoms. It was pre-tested, administered through Google Forms (Google, Mountain View, CA), and available for four weeks. Data were analyzed using descriptive statistics and cross-tabulations with SPSS 26.0 (IBM Corp., Armonk, NY).

Results: The study included 400 participants, with 273 females (68.3%) and 127 males (31.8%). Most participants were single (245, 61.3%) and held a university degree (301, 75.3%). Daily phone use varied: 228 participants (57.0%) used their phones for less than five hours daily, while 43 (10.8%) used them for 10-15 hours or more. Neck positions ranged from 0° to 60°, with 168 participants (42.0%) maintaining a 30° angle. Awareness of health risks associated with phone use was high, with 364 participants (91.0%) aware of these risks. Neck pain was reported by 244 participants (61.0%), with additional symptoms including headache (22 participants, 5.5%) and upper back pain (five participants, 1.3%).

Conclusion: This study found a significant link between prolonged phone use and neck pain in the Eastern Province of Saudi Arabia. Despite high awareness of the risks, many individuals report discomfort. These findings underscore the need for public health interventions and ergonomic education to improve phone use practices and musculoskeletal health.

## Introduction

In recent years, the ubiquitous use of mobile phones has prompted significant interest in understanding their impact on physical health [1,2]. As mobile devices become increasingly integrated into daily life, concerns have arisen regarding their potential effects on musculoskeletal health, particularly about neck pain [3]. This issue is of particular relevance in regions with high mobile phone penetration, such as the Eastern Province of Saudi Arabia, where the use of these devices is prevalent across various demographic groups.

Neck pain is a common complaint among individuals who frequently use mobile phones. The ergonomic challenges associated with prolonged phone use, such as sustained neck flexion and poor posture, are thought to contribute to this issue [2-4]. Research has demonstrated that extended periods of phone use can lead to discomfort and musculoskeletal disorders, including neck pain and stiffness [5,6]. However, there is limited data on the prevalence and severity of these symptoms in specific populations, including those in the Eastern Province of Saudi Arabia.

The Eastern Province, with its rapidly growing population and high levels of mobile phone usage, provides a unique context for examining these concerns. The region's demographic diversity, combined with its high technology adoption rates, makes it an ideal setting for investigating the relationship between phone use and neck pain. Understanding the patterns of phone use and their potential impacts on health in this context can provide valuable insights for public health initiatives and ergonomic recommendations [2-6].

Despite growing awareness of the potential harms associated with prolonged phone use, there is a gap in the literature regarding the specific impact on residents of the Eastern Province. Previous studies have highlighted associations between phone use and musculoskeletal symptoms, but many have focused on different regions or populations [7-16]. This study aims to fill this gap by providing a comprehensive assessment of phone use patterns, neck positions, and associated symptoms among individuals in the Eastern Province.

## Materials and methods

Study design

This cross-sectional study was designed to explore the relationship between phone use and neck pain among residents of the Eastern Province of Saudi Arabia. An online, self-administered survey was used to collect data on various factors, including demographic characteristics, phone use patterns, neck positions during phone use, awareness of potential health risks, and pain symptoms.

Study participants

Participants were recruited using a convenience sampling method. The survey was distributed through multiple online platforms, including social media, local community groups, and university networks, to reach a broad and diverse sample of adults aged 18 years or older residing in the Eastern Province. Individuals with significant cognitive impairments or those unable to complete the survey in Arabic or English were excluded from participation.

Inclusion and exclusion criteria

Participants were included if they were 18 years or older, resided in the Eastern Province of Saudi Arabia, and could complete the survey in Arabic or English. Those with significant cognitive impairments or who could not complete the survey in the required languages were excluded. Incomplete responses were also excluded from the analysis.

Sample size calculation

The sample size was determined to ensure the study's findings were statistically reliable. Based on a 95% confidence level, a 5% margin of error, and an estimated proportion of 50%, the required sample size was approximately 384. To account for non-responses, the final sample size was set at 400 participants.

Survey instrument

The survey instrument was meticulously developed based on a thorough review of existing literature and aimed to capture a comprehensive range of data relevant to the study objectives [16]. The first section gathered demographic information, including gender, marital status, education level, and nationality. This information was crucial for understanding the distribution of respondents across different social and educational backgrounds. The second section focused on phone use patterns. Participants were asked about their average daily phone usage, categorized into intervals such as less than five hours, five to 10 hours, 10 to 15 hours, and more than 15 hours per day. Additionally, the survey inquired about the typical neck position maintained while using the phone, with options ranging from 0° to 60° angles. The third section assessed participants' awareness and perception of potential health risks associated with prolonged phone use. Respondents were asked whether they were aware of these risks and their personal views on the relationship between phone use and neck pain. The final section collected data on symptoms experienced by participants, including neck pain, neck stiffness, hand numbness, headache, upper back pain, and arm pain. Participants were asked to indicate the presence and severity of these symptoms about their phone use (Appendix).

Before its official launch, the survey was pre-tested with a sample of 20 individuals to ensure that questions were clear and relevant. The final survey was administered online using Google Forms (Google, Mountain View, CA), which facilitated secure data collection and ease of access for participants.

Data collection and analysis

The online survey was available for four weeks. During this time, participants accessed the survey via a secure web link. To increase response rates, reminders were sent at the midpoint and toward the end of the data collection period. The design of the survey ensured it was user-friendly, with clear instructions and logical branching to minimize respondent burden and enhance the accuracy of the collected data. Data were analyzed using descriptive statistics to summarize participant characteristics and survey responses. Frequencies and percentages were calculated for categorical variables.

## Results

Study population characteristics

The study included 400 participants, with 68.3% being female and 61.3% being single. A significant majority (75.3%) held a university degree. Detailed demographic characteristics are presented in Table 1.

**Table 1:** Study population characteristics. Frequencies are presented as numbers and percentages (%) of the total sample (n = 400). Percentages are rounded to the nearest whole number and may not sum to 100% due to rounding. Data reflect the demographic characteristics of the study population surveyed in the Eastern Province of Saudi Arabia.

Characteristic		Frequency (n)	Percent (%)
Gender	Female	273	68.3
	Male	127	31.8
Marital status	Single	245	61.3
	Married	150	37.5
	Divorced	4	1.0
	Widowed	1	0.3
Education level	Elementary	3	0.8
	Secondary	80	20.0
	University	301	75.3
	Postgraduate	8	2.0
	Intermediate	8	2.0
Nationality	Saudi	383	95.8
	Non-Saudi	17	4.3

Phone use and neck position

Daily phone use varied among participants, with 57.0% using their phones for less than five hours per day, and 10.8% using them for 10-15 hours. The most common neck position maintained during phone use was 30°, reported by 42.0% of participants. Full distributions of phone use and neck positions are detailed in Table 2.

**Table 2:** Phone use and neck position. Frequencies are presented as numbers and percentages (%) of the total sample (n = 400). Percentages are rounded to the nearest whole number and may not sum to 100% due to rounding. The data represent the distribution of phone use duration and neck positions among participants surveyed in the Eastern Province of Saudi Arabia.

Characteristic		Frequency (n)	Percent (%)
Daily phone use	<5 hours/day	228	57.0
	5-10 hours/day	113	28.2
	10-15 hours/day	43	10.8
	>15 hours/day	16	4.0
Neck position	0°	15	3.8
	15°	85	21.3
	30°	168	42.0
	45°	113	28.2
	60°	19	4.8

Awareness and perception of harm

A large majority (91.0%) of participants were aware of the potential harms associated with prolonged phone use. In terms of perceptions, 77.8% agreed or strongly agreed that phone use contributes to neck pain. Table 3 provides a breakdown of participants' awareness and perceptions.

**Table 3:** Awareness and perception of harm. Frequencies are presented as numbers and percentages (%) of the total sample (n = 400). Percentages are rounded to the nearest whole number and may not sum to 100% due to rounding. The table shows participants' awareness of potential harms and their perception of neck pain associated with phone use based on survey data from the Eastern Province of Saudi Arabia.

Characteristic		Frequency (n)	Percent (%)
Aware of harms	No	36	9.0
	Yes	364	91.0
Perceived neck pain	Strongly disagree	31	7.8
	Disagree	13	3.3
	Neutral	45	11.3
	Agree	145	36.3
	Strongly agree	166	41.5

Perceived pain in different body parts

Neck pain was the most frequently reported symptom, with 77.8% of participants agreeing or strongly agreeing that they experienced neck pain related to phone use. Other reported symptoms included headaches (reported by 75.0% of participants) and upper back pain (67.6%). Table 4 details the frequency of reported symptoms.

**Table 4:** Perceived pain in different body parts. Frequencies are presented as numbers and percentages (%) of the total sample (n = 400). Percentages are rounded to the nearest whole number and may not sum to 100% due to rounding. The table details participants' perceptions of pain in various body parts based on survey responses from the Eastern Province of Saudi Arabia.

Body part		Frequency (n = 400)	Percent (%)
Neck pain	Strongly disagree	31	7.8
	Disagree	13	3.3
	Neutral	45	11.3
	Agree	145	36.3
	Strongly agree	166	41.5
Arm pain	Strongly disagree	41	10.3
	Disagree	33	8.3
	Neutral	67	16.8
	Agree	133	33.3
	Strongly agree	126	31.5
Shoulder pain	Strongly disagree	35	8.8
	Disagree	32	8.0
	Neutral	68	17.0
	Agree	126	31.5
	Strongly agree	139	34.8
Upper back pain	Strongly disagree	34	8.5
	Disagree	35	8.8
	Neutral	61	15.3
	Agree	123	30.8
	Strongly agree	147	36.8
Headache	Strongly disagree	27	6.8
	Disagree	19	4.8
	Neutral	54	13.5
	Agree	124	31.0
	Strongly agree	176	44.0

Symptoms experienced and neck pain using the phone

Among participants, 61.0% reported experiencing neck pain while using their phones. Additional symptoms, such as headaches (5.5%) and upper back pain (1.3%), were also reported. These relationships are summarized in Table 5.

**Table 5:** Symptoms experienced and neck pain while using a phone. Frequencies are presented as numbers and percentages (%) of the total sample (n = 400). Percentages are rounded to the nearest whole number and may not sum to 100% due to rounding. The table shows the relationship between neck pain reported during phone use and other symptoms experienced by participants surveyed in the Eastern Province of Saudi Arabia.

Characteristic		Frequency (n)	Percent (%)
Neck pain while using a phone	No	156	39.0
	Yes	244	61.0
Experienced symptoms	Neck pain	11	2.8
	Neck stiffness	1	0.3
	Hand numbness	1	0.3
	Headache	22	5.5
	Upper back pain	5	1.3
	Arm pain	5	1.3

## Discussion

This study aimed to investigate the relationship between phone use and neck pain among residents of the Eastern Province of Saudi Arabia. Our findings indicate that a significant proportion of participants reported experiencing neck pain related to their phone use. Specifically, 61.0% of respondents reported neck pain while using their phone, with a notable prevalence of other musculoskeletal symptoms such as headaches, upper back pain, and arm pain.

The majority of participants were aware of the potential harms associated with prolonged phone use, which is consistent with existing literature highlighting growing public awareness of such risks. However, despite this awareness, many individuals continue to engage in behaviors that could exacerbate neck pain, such as maintaining awkward neck positions for extended periods. This suggests a disconnect between knowledge and behavior, which may be influenced by the high demand and convenience of mobile phone use [8-11].

Our study aligns with previous research demonstrating a link between prolonged phone use and neck pain. Studies conducted in other regions have similarly found that extensive use of mobile phones can lead to musculoskeletal issues, particularly in the neck and upper back areas. For instance, research from various countries has highlighted that sustained neck flexion and poor posture while using phones contribute to discomfort and pain [7-12]. Our findings extend these observations to the specific context of the Eastern Province, adding regional insights to the global conversation on this issue.

The high prevalence of neck pain and other related symptoms among our study participants underscores the need for targeted interventions and public health strategies. Employers, educators, and health professionals in the Eastern Province should consider incorporating ergonomic education and posture correction strategies into their practices. Additionally, public health campaigns that emphasize the importance of regular breaks and proper posture while using mobile devices could help mitigate the risk of musculoskeletal disorders [11-15].

Recent research conducted in Riyadh, Saudi Arabia, further corroborates the high prevalence of musculoskeletal symptoms linked to extensive smartphone use. The study by Abu Halimah et al. [16] highlights a significant occurrence of neck pain (81.1%), headaches (63.0%), and shoulder pain (49.4%) among smartphone users, reflecting concerns similar to those observed in other regions. This aligns with global findings that suggest prolonged smartphone usage contributes to musculoskeletal disorders such as text neck syndrome, exacerbated by poor posture and extended screen time.

Several limitations should be acknowledged in interpreting the results of this study. The cross-sectional design of the survey limits our ability to establish causality between phone use patterns and neck pain. Additionally, the reliance on self-reported data may introduce bias, as participants might underreport or overreport symptoms based on their perceptions. The convenience sampling method, while useful for reaching a broad audience, may also limit the generalizability of the findings to other regions or populations.

## Conclusions

This study reveals a significant association between prolonged phone use and neck pain among residents of the Eastern Province of Saudi Arabia, shedding light on an important public health issue in the region. Despite a high level of awareness regarding the potential risks associated with excessive phone use, a substantial proportion of the population continues to experience discomfort and musculoskeletal symptoms, particularly neck pain. This discrepancy between awareness and behavior suggests that knowledge alone may not be sufficient to change habits, highlighting the complexity of addressing such health concerns in a technology-driven society.

The findings underscore the urgent need for targeted public health interventions that go beyond merely raising awareness. These interventions should aim to translate knowledge into practice by promoting ergonomic education and encouraging behavioral changes that reduce the risk of musculoskeletal disorders. Educational programs that emphasize proper posture, the importance of regular breaks, and the use of ergonomic devices could play a crucial role in mitigating the adverse effects of prolonged phone use. By addressing these issues proactively, it is possible to not only alleviate current discomfort and pain but also prevent more severe musculoskeletal problems from developing in the future. As smartphone usage continues to grow globally, the insights gained from this study could inform strategies that promote healthier technology use, ultimately enhancing the quality of life for individuals in the Eastern Province and beyond.

## Appendices

Survey questionnaire

Section 1: Demographic Information

Age: Under 18 | 18-25 | 26-30 | 31-40 | 41-50 | Above 50

Gender: Male | Female | Other

Marital status: Single | Married | Divorced | Widowed

Education level: Primary | Intermediate | High school | College | Higher studies

Nationality: Saudi | Non-Saudi

Section 2: Smartphone Usage

Daily smartphone use: 0-2 hours | 2-3 hours | 3-5 hours | More than 5 hours

Typical neck position: 0 degrees | 15 degrees | 30 degrees | 45 degrees | 60 degrees

Awareness of health effects: Yes | No

Section 3: Awareness and Symptoms

Heard of "text neck" syndrome? Yes | No

Source of information about text neck: Friends/relatives | Internet | Doctors | Medical journals | Other

Diagnosed with text neck syndrome? Yes | No

Section 4: Musculoskeletal Symptoms

Symptoms severity:

Neck pain: No pain | Mild | Severe | Very severe

Arm pain: No pain | Mild | Severe | Very severe

Shoulder pain: No pain | Mild | Severe | Very severe

Upper back pain: No pain | Mild | Severe | Very severe

Headaches: No headache | Little | Moderate | Severe

Stiff neck: Never | Rarely | Occasionally | Frequently | Always

Hand numbness: No numbness | Mild | Moderate | Severe
